# Screening of Acrylamide of Par-Fried Frozen French Fries Using Portable FT-IR Spectroscopy

**DOI:** 10.3390/molecules27041161

**Published:** 2022-02-09

**Authors:** Didem P. Aykas, Alejandra Urtubia, Kevin Wong, Luju Ren, Claudia López-Lira, Luis E. Rodriguez-Saona

**Affiliations:** 1 Department of Food Engineering, Faculty of Engineering, Adnan Menderes University, Aydin 09100, Turkey; didem.cinkilic@adu.edu.tr; 2 Department of Food Science and Technology, The Ohio State University, 100 Parker Food Science and Technology Building, 2015 Fyffe Road, Columbus, OH 43210, USA; alejandra.urtubia@usm.cl (A.U.); wong.687@buckeyemail.osu.edu (K.W.); ren.276@buckeyemail.osu.edu (L.R.); clopez6@uc.cl (C.L.-L.); 3 Department of Chemical and Environmental Engineering, Universidad Técnica Federico Santa María, Av. España 1680, Valparaíso 2390123, Chile

**Keywords:** French fries, acrylamide, QuEChERS, vibrational spectroscopy, HPLC-MS/MS

## Abstract

Current assays for acrylamide screening rely heavily on LC-MS/MS or GC-MS, techniques that are not suitable to support point of manufacturing verification because it can take several weeks to receive results from a laboratory. A portable sensor that can detect acrylamide levels in real-time would enable in-house testing to safeguard both the safety of the consumer and the economic security of the agricultural supplier. Our objective was to develop a rapid, accurate, and real-time screening technique to detect the acrylamide content in par-fried frozen French fries based on a portable infrared device. Par-fried French fries (*n* = 70) were manufactured at times ranging from 1 to 5.5 min at 180 °C to yield a wide range of acrylamide levels. Spectra of samples were collected using a portable FT-IR device operating from 4000 to 700 cm^−1^. Acrylamide was extracted using QuEChERS and quantified using uHPLC-MS/MS. Predictive algorithms were generated using partial least squares regression (PLSR). Acrylamide levels in French fries ranged from 52.0 to 812.8 µg/kg. The best performance of the prediction algorithms required transformation of the acrylamide levels using a logarithm function with models giving a coefficient of correlation (Rcv) of 0.93 and RPD as 3.8, which means the mid-IR model can be used for process control applications. Our data corroborate the potential of portable infrared devices for acrylamide screening of high-risk foods.

## 1. Introduction

Even though French fries are considered unhealthy foods, they are one of the most popular and palatable foodstuffs for people, and large quantities are consumed worldwide, especially by young people [1]. The high content of salt and fat is not the only concern about the French fries, but also its preparation process. Food items, particularly containing carbohydrates, that are fried, baked, or roasted at a higher temperature (above 120 °C) generate acrylamide, which is a harmful compound formed through the Maillard reaction pathway [2,3,4,5].

Acrylamide has been reported as neurotoxic [6] and as a potential carcinogen to humans (Group 2A) based on its carcinogenicity in rats [6,7,8,9]. Olesen and others [10] found that the cancer risk level depends on the percentage of the acrylamide present in the potato chips, depending on how long and at what temperature they are fried. Research has found that French fries and potato chips baked or fried at high temperatures (>180 °C) and for long durations contain higher acrylamide amounts than the safe levels for consumption [11]. Romani and others [12] reported that increasing frying time causes an exponential increase in acrylamide level and its formation rate. Additionally, Jackson and Al-Taher [13] reported that the highest acrylamide content was associated with the most processed sample, which undergoes the highest frying time and highest frying temperature; in addition, that sample showed the highest degree of dark color since there is a positive correlation between the brown color formation and acrylamide levels [13].

Governments have taken steps to protect the public from carcinogens in the food supply. For instance, Proposition 65 in California requires that all products that contain potential carcinogens, including acrylamide, bear a warning label. Companies are required to keep acrylamide levels below 275 µg/kg to avoid having a warning label on their products [14]. However, the Food Drug Administration has not set maximum levels for acrylamide in foods; instead, it has decided to take on mitigation strategies and monitoring procedures [15].

High concentrations of acrylamide in French fries and potato chips have been reported at levels of 30–2300 µg/kg [16], 306–775 µg/kg [17], or even more than 4000 µg/kg in crisps [1]. Furthermore, a study has shown that French fries and potato chips may contribute up to 56% of the total acrylamide intake in the Western diet of adolescents [18]. Therefore, it is highly necessary to monitor the acrylamide formation and levels throughout the production, and mitigation approaches should be practiced, including employing good manufacturing practices and the application of measures based on hazard analysis and critical control point principles, selecting potato varieties with lower sugar content, suitable storage and transportation, suppressing sprouting, blanching, the addition of disodium diphosphate, treatment with asparaginase, increasing French fry thickness, and lowering the frying temperature [1,19].

Although current analytical methods based on chromatography in tandem with mass spectroscopy detection can accurately measure acrylamide content in foods, they are not suitable to support the point of manufacturing verification and can hardly be implemented as routine analysis at food companies. Infrared (IR) spectroscopy is a rapidly improving technology that has been applied by the food industry for quality and safety screening purposes. It has many advantages, such as a low upkeep cost, rapid analysis time, high throughput, and little to no training required for the operator. Fourier transform infrared (FT-IR) spectroscopy combined with chemometrics provides a robust and rugged technique that can be used to determine contaminants in foods. Portable IR instruments can provide quality control technicians with a highly mobile method for screening products off the line with minimal sample preparation. Moreover, the miniaturization of vibrational spectroscopy equipment allows on-site and real-time monitoring of food products and production processes to ensure quality and safety [20]. Nonetheless, few studies have reported the use of infrared spectroscopy to determine acrylamide in potato chips and French fries [21,22,23], and further research is needed to establish infrared spectroscopy as a screening tool of acrylamide content in foods.

Our objective was to develop a simple screening technique based on a portable mid-infrared (mid-IR) instrument to detect the acrylamide content in par-fried frozen French fries with minimal sample preparation.

## 2. Results and Discussion

Acrylamide levels in par-fried frozen French fries were determined by quantifying the major ions (72 > 55 *m*/*z*) using LC-MS/MS and were found to range from 52.0 to 812.8 µg/kg, which is consistent with values reported in the literature [1,24,25].

**Figure 1:**
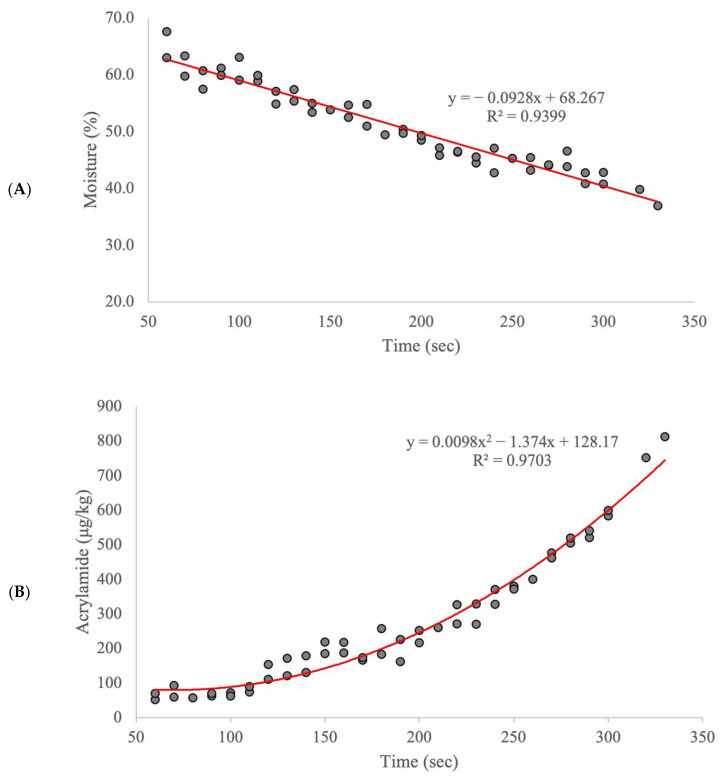

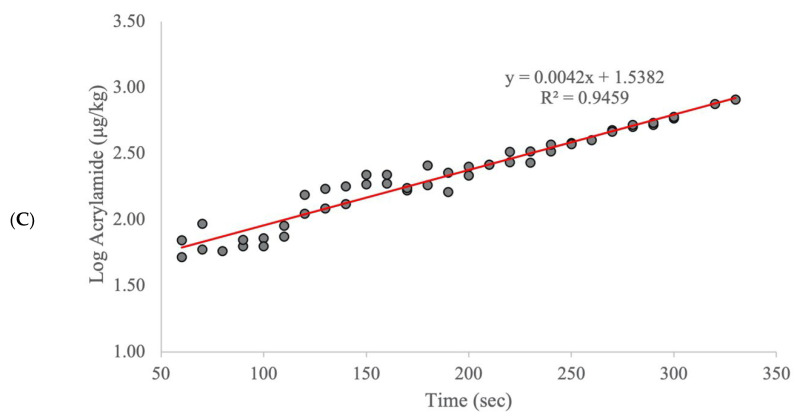
Plots for the (**A**) moisture content in French fries over the frying time, (**B**) average acrylamide concentration measure by LC-MS/MS against the frying time, and (**C**) logarithm transformation of the acrylamide concentration against the frying time.

The total solid content in par-fried frozen French fries ranged from 32.4 to 63.0%. A similar total solid content in the French fry samples was reported in the literature [26]. The moisture loss in the samples increased with frying time and followed zero-order kinetics (Figure 1A). The acrylamide content in the samples increased with frying time (one to five and a half minutes) following a pseudo-first-order reaction rate (Figure 1B). The exponential increase in acrylamide formation with the increase in frying time has been previously reported by other researchers [12,27,28,29]. One interesting finding is that the plot does not intersect at zero, indicating that there may be a nonzero minimum level of acrylamide formation the instant that potatoes come into contact with hot oil. Figure 1C shows the logarithm transformation of the first-order reaction rate of the acrylamide content, in which the log values of acrylamide concentration were plotted against frying time, giving a linear relationship between the two parameters.

**Figure 2:**
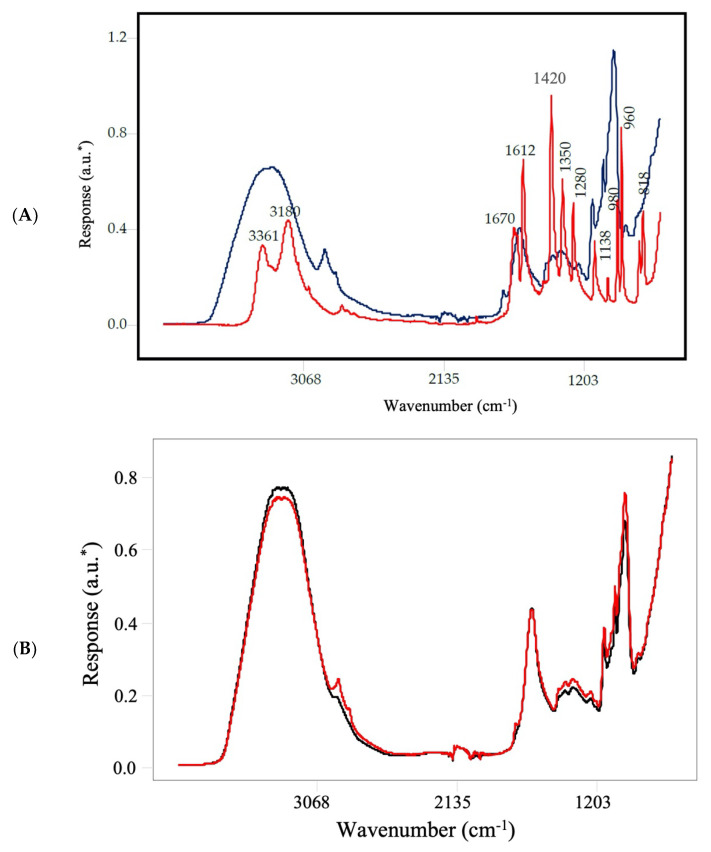
Representative spectra of a French fry powder and an acrylamide standard collected using portable FT-IR spectrometer. * a.u. arbitrary units. The black line represents the French fry powder spectra; the red line represents the acrylamide standard spectra (**A**). Comparison of the representative raw spectrum of French fry samples par-fried for 1 min (black line) and 4 min (red line). * a.u. arbitrary units (**B**).

Typical FT-IR spectra obtained in the mid-IR region (4000–700 cm^−1^) of a powdered French fry sample and an acrylamide standard are shown in Figure 2A. The mid-infrared region provided high resolution in showing distinct and discernable bands for the French fry sample and the acrylamide standard. Several distinct bands were in the spectra that are associated with specific functional groups. The broadband at around 3600–3000 cm^−1^ results from the strong water absorption in the French fry sample, with the maximum of the OH stretching band at around 3300 cm^−1^ [30]. On the other hand, in the same region, the pure acrylamide standard showed more distinct bands when compared to the French fry sample as the standard has limited water content. The bands at around 3360 and 3180 cm^−1^ are assigned to the N–H valence stretching vibration from the cross-linking bridges and the stretching of the hydroxyl (O–H) groups [31,32]. In the sample, the band at around 1746–1720 cm^−1^ represents the C=O functional group, both associated with lipids. The bands associated with the peptide linkage can be seen at around 1690–1600 cm^−1^ for amide I, 1575–1480 cm^−1^ for amide II, and 1301–1229 cm^−1^ for amide III [33]. Finally, the bands located around 1200–900 cm^−1^ linked to the C–O and C–C stretching, as well as the C–O–H and C–O–C deformation of carbohydrates [34]. Figure 2A also shows the FT-IR spectrum of the acrylamide standard, featuring bands centered at 3350 and 3160 cm^−1^ corresponding to N–H stretching frequencies, and the bands at 1670 and 1612 cm^−1^ attributed to C–O and C=C vibrations, respectively. The region in 1400–1100 cm^−1^ is related to the deformation vibrations of –C–N– links and CH_2_ groups. Thus, the bands at around 1420 and 1350 cm^−1^ are associated with C–N stretching and NH_2_ scissoring frequencies, respectively. Finally, the bands centered at 1138 cm^−1^ and 980 cm^−1^ are associated with the vibration of C–C and C=C–H, respectively [31,32].

The visual comparison of FT-IR spectra for par-fried French fries for 1 min and 4 min are given in Figure 2B. The moisture loss in the French fry samples with increasing frying time can be observed from the spectra (3600–3000 cm^−1^). With increasing frying time, the bands associated with oil content increased over time [35] and the bands centered at 2925 and 2854 cm^−1^ attributed to =C–H *cis* stretching and asymmetric and symmetric stretching vibrations of CH_2_ and CH_3_ were more prominent, respectively. Furthermore, it can be observed an increase in the absorbance at 1746 cm^−1^, assigned to –C=O ester stretching vibration, and commonly used in determining the total lipid content [36]. Lastly, increasing the par-frying time enhanced the absorption of carbohydrate bands (~1200–900 cm^−1^). As the acrylamide content on the French fry samples was at ppb levels (52.0 to 812.8 µg/kg), there was not a clear marker band for acrylamide that can be visualized in the spectra (Figure 2B), and the differences in the acrylamide levels were revealed by the chemometric analysis.

Table 1 summarizes the performance statistics of the PLSR models developed using the FT-IR sensor. The infrared spectrometer gave excellent performances, with a high coefficient of correlation (R = 0.93) and a low standard error of cross-validation (SECV = 58.7 µg/kg) (Table 1). Using the leave-one-out cross-validation approach, we selected seven latent factors that explain the systematic variation in the model and produced the minimum value of the root-mean-squared error of cross-validation. While generating the calibration model, a total of four samples were determined as an outlier and excluded from the model. Therefore, 53 samples were used to generate the calibration model, and the remainder (*n* = 13) were used in the external validation model.

**Figure 3:**
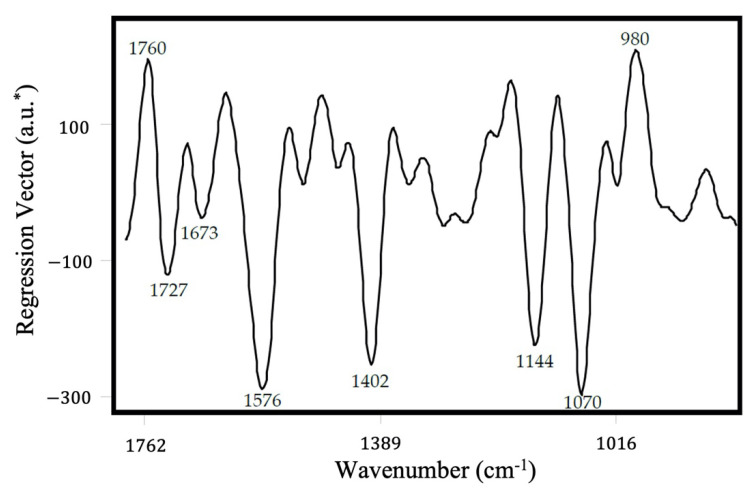
PLSR regression vector plot of the model generated using portable FT-IR spectrometer.

The regression vector plot (Figure 3) helps visualize the model’s highest relevant variation; in other words, it demonstrates the most influential mid-IR bands/regions toward developing the model. Accordingly, the highest relevant variation to estimate the acrylamide content in the training set samples was in the range of 1780 to 980 cm^−1^. The regression vector plot (Figure 3) indicates that some unique features in the vectors matched the pure acrylamide spectra (Figure 2), in particular, the band at 1673 cm^−1^ assigned to the C–O vibration, the signal corresponding to double C=C bonds of the acrylamide end group (1575 cm^−1^), the C–N stretching observed at around 1400 cm^−1^, and the vibration of H in C=C–H observed at 980 cm^−1^ [31,32,37]. In addition, the regression vector also showed the influence of the French fries, with the frequency at about 1140 cm^−1^ indicating the presence of glycosidic C–O–C bonds, while the band at 1070 cm^−1^ can be assigned to the crystalline regions of starch [38].

The calibration model was externally validated using a new set of samples to evaluate the prediction performance of the models. The performance statistics for both models (calibration and external validation) were similar in terms of correlation coefficients (R_CV_–R_Pre_) and standard errors or the models (SECV–SEP) (Table 1), which suggests that when the calibration model encounters new samples in the future, it will successfully predict the acrylamide content of those new French fry samples. Furthermore, we calculated the RPD for models generated with mid-IR data and gave the value of 3.8 (Table 1), indicating that the generated model can be used for process control applications.

**Figure 4:**
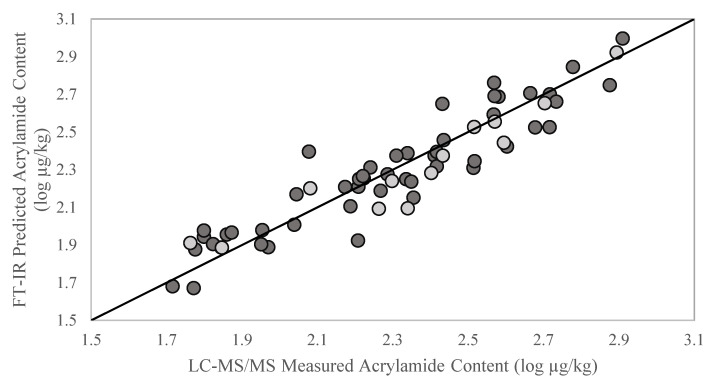
Partial least squares regression (PLSR) plots for acrylamide content in French fry samples using portable FT-IR spectrometer. The dark grey circles represent the calibration set samples and the light grey circles represent the external validation set samples.

The PLSR plot (Figure 4) was generated to visually inspect the distribution of the samples over the regression line. In that plot, both the calibration (dark grey diamonds) and external validation (white diamonds) datasets were represented, and their similar distribution was observed.

Variance-based modeling can be distorted by hidden collinearity in the dataset. By employing multivariate approaches such as PLSR, the regression algorithm minimizes the effects of variable collinearity and model overfitting by checking for redundancy. During the par-frying process, the French fries uptake oil and lose water, which could add hidden collinearity in the analysis. Moyano and Pedreschi [39] reported that the oil uptake in potato slices can be described by fitting a first-order kinetics model. Major differences in the kinetics of moisture loss (zero-order), oil uptake (first-order), and formation of acrylamide (pseudo-first-order) minimize the effect of correlation values among these components. Furthermore, PLSR models provided adequate predictive ability when we excluded the carbonyl (1746 cm^−1^) and carbohydrate (1200–700 cm^−1^) regions (data not shown); however, the models performed better for the validation set when we widened the spectral range from 1800 to 900 cm^−1^, indicating that the training model was enriched by including the variance associated with lipids and carbohydrate variables. The regression vector indicated the importance of bands associated with acrylamide functional groups (1576 and 1400 cm^−1^).

Furthermore, to provide additional comparison with the literature data, SECV and SEP values were also calculated using raw data (logarithmic conversion was not applied). The SECV value was calculated as 58.7 μg/kg and the SEP value was calculated as 55.1 μg/kg. Overall, the calibration and external validation models that we obtained in this study for the determination of acrylamide content using the portable FT-IR sensor provided superior performance in terms of R and SECV or SEP compared to the other studies in the literature conducted on potato products and using benchtop or portable/handheld infrared instruments. Ayvaz and Rodriguez-Saona [22] reported using portable and handheld infrared devices for screening acrylamide in commercial potato chip samples, reporting R_Pre_ > 0.90 and SEP < 100 μg/kg using 6 to 8 factors, with similar performances of the portable and benchtop systems. In the literature, in addition to the FT-IR sensors, NIR sensors have also been applied to determine acrylamide content. Segtnan and others [40] modeled the acrylamide content by creating a range of acrylamide concentrations on a single variety of potato tuber using different frying times and temperatures. Acrylamide contents were predicted by combining the benchtop NIR spectra with reference data collected by liquid chromatography high-resolution mass spectroscopy (LC-HRMS). In this study, the best PLSR performances were obtained using three factors, and the prediction error was determined as 247 μg/kg with a coefficient of correlation of 0.95. In a study conducted by Pedreschi and others [21] on potato chips, the acrylamide content was predicted with an average prediction error of 266 μg/kg and R = 0.83 using an online VIS/NIR interactance line scanner. Adedipe and others [23] evaluated a benchtop NIR system to predict acrylamide content in French fries produced using the Russet Norkotah variety and reported a SEP of 135 μg/kg and R_Pre_^2^ = 0.97 using nine factors and the spectral wavelength range of 1100−2500 nm.

Even though sensitivity challenges have been reported using FT-IR spectroscopy, advances in instrumentation and multivariate data analysis techniques make the FT-IR technology able to detect minor food components down to parts-per-billion (ppb) levels [41]. The mentioned ppb level has detected food components including acrylamide in potato chips [22], tetracycline in milk [42], determination of chlorinated hydrocarbons in water [43], levels of organic pollutants in the aquatic environment (i.e., aromatics, alkyl halides, phenols) [44], and mycotoxins [45]. In our study, the increased sensitivity comes from the enhanced evanescent field by the ATR 3-reflection crystal that is in close contact with the sample rather than the sensor’s sensitivity [43,44], which improves the signal-to-noise ratio and allows maximizing the fingerprinting capabilities of the FT-IR instrument.

## 3. Materials and Methods

### 3.1. French Fry Material

A total of 70 par-fried frozen French fries were kindly provided by a leading manufacturer (McCain Foods, New Brunswick, Canada). Samples were fried in corn oil at 180 °C for one to five and a half minutes, and samples (100 g) were pulled at 10 s intervals. A fresh batch of oil was used for par-frying the French fries to prevent any compositional changes associated with rancidity. French fry samples were immediately frozen and overnight-shipped in insulated boxes with dry ice to the Department of Food Science and Technology at The Ohio State University. The frozen French fry samples were blended using a Waring blender (East Windsor, NJ, USA) until having a fine powder. Sample blending was performed to ensure a homogenous sample representing each batch of French fries. Powdered French fry samples were used for LC-MS/MS analysis and FT-IR spectral collection. Samples were randomly divided into the calibration/training and external validation model. In the calibration/training model, 80% of the total samples were used, and in the external validation model, the remaining 20% of the total samples were used. All samples (both the calibration and the external validation) were analyzed by LC-MS/MS and FT-IR spectroscopy within two weeks after receiving the samples. Samples were stored in a freezer (−40 °C) to protect them from any changes until the analysis.

### 3.2. Acrylamide Extraction and LC-MS/MS Analysis

The QuEChERS (Quick, Easy, Cheap, Effective, Rugged, and Safe) method was used to extract acrylamide from the frozen French fry powder. The method used for this study followed the experiment outlined by [46]. Extraction and dispersive QuEChERS kits were obtained from Agilent Technologies (Santa Clara, CA, USA) to extract and purify French fry samples. LC-MS/MS analysis was performed in two independent sets of French fry samples. The French fry powder (1 g) was placed into a 50 mL centrifuge tube and spiked with 0.5 mL of a 1 ppm solution of ^13^C-labeled acrylamide internal standard in acetonitrile (Acros Organics, Fairlawn, NJ, USA). Hexane (5 mL) was added to the tube and vortexed for 30 s to remove the fat from the sample. Then, 10 mL of water, 10 mL of acetonitrile, and a salt mixture containing 4 g of MgSO_4_ and 0.5 g of NaCl were added to each sample, shaken vigorously for one minute, and centrifuged for 5 min at 5000 rpm. The hexane layer was discarded, and 1 mL of the acetonitrile layer was transferred into a 2 mL microcentrifuge vial containing 50 mg of primary secondary amine (PSA) and 150 mg of MgSO_4_. The microcentrifuge vial was vortexed for 30 s and centrifuged for 1 min at 5000 rpm, and the supernatant was filtered through a 0.45 µm nylon filter and transferred into an autosampler vial for LC-MS/MS analysis.

A standard curve using acrylamide (99%+) and ^13^C-labeled acrylamide (Acros, Fairlawn, NJ, USA) was created to quantify the acrylamide concentration in extracts via mass spectroscopy. The concentration of ^13^C-labeled acrylamide was kept constant throughout the standard curve. A stock solution with an initial concentration of 1000 µg/kg was serially diluted to obtain an acrylamide range from 7.8 to 1000 µg/kg.

A Shimadzu Nexera-I LC-2040C uHPLC (Kyoto, Kyoto Prefecture, Japan) equipped with a Shimadzu LCMS-8040 tandem mass spectrometry detector (Kyoto, Kyoto Prefecture, Japan) was used for the analysis. A Pinnacle DB C18 column (50 × 2.1 mm with a particle size of 1.9 um) manufactured by Restek (Bellefonte, PA, USA) was used for the analysis. The instrument ran under positive electrospray mode with an oven temperature of 40 °C. The nebulizing gas was run at a rate of 1.5 L/min. The transition *m*/*z* was taken at 72 > 55 for acrylamide and 75 > 58 for ^13^C-labeled acrylamide. The collision energy used by the tandem mass spectrometer was −31 V and a multiple ion monitoring mode was used. A solvent of 0.1% formic acid in water was applied isocratically at a flow rate of 0.3 mL/min.

### 3.3. FT-IR Spectroscopy

A portable FT-IR spectrometer (4500a, Agilent Technologies, Santa Clara, CA, USA) was used to collect mid-infrared (mid-IR) spectra. The instrument is equipped with a three-reflection diamond ATR accessory along with a deuterated triglycine sulfate (DGTS) detector and uses a Michaelson interferometer to disperse light. A uniform pressure was applied to the sample using the provided apparatus for sampling solids. The FT-IR spectra were collected from the frequency range 4000–700 cm^−1^, using 64 background scans and 64 sample scans to improve the signal-to-noise ratio. The spectral resolution was set to 4 cm^−1^ and an air background was collected before each spectral collection to account for environmental variations. Two independent FT-IR spectra were collected from powdered French fry samples and the average spectra were used for chemometric analysis.

### 3.4. Chemometric Analysis

The acrylamide content in the French fries’ quantification model was generated using a multivariate technique known as partial least squares regression (PLSR). PLSR correlates two data matrices (FT-IR spectra and the acrylamide concentrations obtained through LC-MS/MS analysis) using a linear multivariate model while maximizing their co-variance. PLSR detects the patterns in large datasets and reduces a large number of variables (the FT-IR spectra consist of thousands of data points) to a set of dependent variables (latent variables/factors) that represent the co-variance between the spectra and the LC-MS/MS data [47,48].

Before the PLSR analysis, the dataset was split into the calibration/training set that consists of 80% of the total sample size, and the external validation set that includes the remaining 20% of the samples. Full cross-validation (leave-one-out approach) was implemented in the calibration set to validate the calibration model internally. The optimum number of LVs that was used in the calibration model was determined by selecting the LV number that results in the first local minimum standard error of cross-validation (SECV). Choosing the optimum LV while generating the calibration model is critical to prevent over- or under-fitting the model. Selecting additional LVs could cause over-fitting of the model that could also lead to modeling random noise [49], or choosing too few LVs could cause under-fitting of the model that eliminates valuable information, essential to explain the model [47]. The external validation set was used to externally validate the calibration model to evaluate its robustness and to determine if the calibration model can predict the new French fry samples’ acrylamide content with a similar performance in the future. In addition, the generated calibration model performance and the goodness of the model fit were evaluated through the standard error of cross-validation (SECV), coefficient of correlation for cross-validation (R_cv_), standard error of prediction for external validation (R_Pre_), and residual predictive deviation (RPD).

The outlier diagnostics (studentized residual of sample vs. leverage) was used to identify any unusual samples and helped to optimize the multivariate model. During the generation of the calibration and external validation models, samples with a large studentized residual (>3) and/or high leverage were re-evaluated and excluded from the model if needed. The RPD is the ratio between the LC-MS/MS reference values’ standard deviation in the calibration set and the SEP. The RPD value indicates how accurately the generated model can predict the interested compound (the acrylamide content) [50]. Accordingly, the higher the RPD value, the higher the power to predict the French fries’ acrylamide levels. RPD values between 0 and 1.9 are not recommended for any application, RPDs between 2.0 and 2.4 provide rough screening capabilities, RPDs between 2.5 and 2.9 allow for screening, RPDs of 3.0–3.4 are good for quality control, RPDs of 3.5–4.0 are suitable for process control applications, and RPDs above 4.1 can be used for any type of application [51].

Pirouette^®^ chemometrics software (Version 4.5 Infometrix Inc., Bothell, WA, USA) was used to correlate the spectral data and reference analysis results. The FT-IR spectra were preprocessed with mean-centering to reduce the micro-multicollinearity, then transformed using the Savitzky–Golay second derivative (35-points window) polynomial filter and smoothing (35-points window) to resolve overlapping signals and exceed the instrument noise. Mean-centering, Savitsky–Golay, and smoothing options were determined and applied after assessing other preprocessing and transformation algorithms, including divide-by (sample 2-norm), normalization, first-derivatives, standard normal variate (SNV), and multiplicative scatter correction (MSC).

## 4. Conclusions

This study evaluated the application of a portable FT-IR sensor with chemometric analysis to predict the acrylamide content in French fry samples. Our results showed that the acrylamide content increases with frying time, and this increase in the acrylamide content followed a first-order reaction rate instead of zero-order kinetics. The PLSR model performance statistics using FT-IR (R_Pre_ = 0.94, SEP = 55.1 μg/kg) demonstrated that the examined portable instrument can accurately predict the acrylamide levels in French fries as that instrument showed a consistent and strong correlation of acrylamide levels. Furthermore, the evaluated sensor has several advantages over traditional techniques (LC-MS/MS and GC-MS), including having a lower upkeep cost, a higher sample throughput, and potential for in-field applications. Our results suggest that the models developed using portable infrared sensors can be used as a reliable screening tool in the snack food industry or the government agencies for acrylamide levels in French fries.

## Figures and Tables

**Table 1:** Statistical performance of the PLSR prediction models for acrylamide content in French fry samples developed using portable FT-IR spectrometer.

Calibration Model					External Validation Model				
Range	N ^a^	Factor	SECV ^b^	R_CV_ ^c^	Range	n ^d^	SEP ^e^	R_pre_ ^f^	RPD ^g^
52.0–812.8	53	7	58.7	0.93	57.9–783.4	13	55.1	0.94	3.8

^a^ Number of samples used in calibration models. ^b^ Standard error of cross-validation. ^c^ Coefficient of correlation of cross-validation. ^d^ Number of samples used in external validation models. ^e^ Standard error of prediction. ^f^ Coefficient of prediction for external validation. ^g^ Residual predictive deviation. SECV and SEP are in units of μg/kg.

## Data Availability

Not applicable.

## Sample Availability

Samples of the compounds are not available from the authors.
